# Covid-19 effects on the workload of Iranian healthcare workers

**DOI:** 10.1186/s12889-020-09743-w

**Published:** 2020-11-02

**Authors:** Esmail Shoja, Vahideh Aghamohammadi, Hadi Bazyar, Hamed Rezakhani Moghaddam, Khadijeh Nasiri, Mohammad Dashti, Ali Choupani, Masoumeh Garaee, Shafagh Aliasgharzadeh, Amin Asgari

**Affiliations:** 1 School of Medical Sciences Esfarayen Faculty of Medical Sciences, Esfarayen, Iran; 2 Department of Nutrition, Khalkhal University of Medical Sciences, Khalkhal, Iran; 3 Student Research Committee, Ahvaz Jundishapur University of Medical Sciences, Ahvaz, Iran; 4 Department of Public Health, Khalkhal University of Medical Sciences, Khalkhal, Iran; 5 Department of Nursing, Khalkhal University of Medical Sciences, Khalkhal, Iran; 6 Department of Ergonomics, Sharif Safety Index, Tehran, Iran; 7 Department of Internal Medicine, School of Medicine and Allied Medical Sciences, Imam Khomeini Hospital, Ardabil University of Medical Sciences, Ardabil, Iran; 8 Department of Environmental Health, Khalkhal University of Medical Sciences, Khalkhal, Iran

**Keywords:** COVID-19, Health worker, Mental health, Workload

## Abstract

**Background:**

In this study, we aimed to evaluate the impact of the COVID-19 epidemic on the workload and mental health of Iranian medical staff using the General Health Questionnaire (GHQ-12) and NASA -Task Load Index (NASA-TLX) Questionnaire between March and April 2020, respectively.

**Methods:**

The present cross-sectional study was conducted from March 5th to April 5th, 2020. To evaluate the workload and mental health of participants NASA-TLX and GHQ-12 online questionnaires were distributed. Data were entered into software SPSS (Version 23) and T-test, ANOVA, Regression methods were used for data analysis.

**Results:**

Health workers who encountered COVID- 19 patients, were subjected to more task load compared to those who had no contact with COVID- 19 patients at the workplace (*p* <  0.001). In terms of the subscale score of NASA-TLX, nurses had more scores in mental pressure, physical pressure, time pressure (temporal), and frustration compared to the other jobs (*p* <  0.05). Moreover, nurses had significantly more workload compared to the other jobs.

**Conclusions:**

Type of job, the shift of work, educational level, and facing COVID-19 affected the score of NASA-TLX. NASA-TLX scores were higher in nursing compared to the scores of other health staff groups. The results of this study indicate that the scores of NASA-TLX and GHQ-12 among staff who had contact with COVID-19 patients were significantly higher than those who did not face COVID-19 patients. We suggested that a comprehensive assistance should be provided to support the well-being of healthcare workers especially nurses and healthcare workers who treated COVID-19 patients.

**Supplementary Information:**

**Supplementary information** accompanies this paper at 10.1186/s12889-020-09743-w.

## Background

The 2019 novel coronavirus (COVID-19) appeared in December 2019, in Wuhan, China. COVID-19 was shown to be caused by SARS-CoV-2, which is a positive-sense single-stranded RNA virus belonging to the subgenus Sarbecovirus (beta-CoV lineage B) [1]. On 30th January 2020, due to the spread of this virus to other countries following a logarithmic growth, WHO stated the outbreak of COVID-19 as a Public Health Emergency of International Concern (PHEIC) [2]. Despite the low mortality rate of that as 2%, the COVID-19 virus has a high transmission rate as well as a higher mortality rate than that caused by both severe acute respiratory syndrome (SARS) and middle east respiratory syndrome (MERS) [3]. In this regard, to reduce the rate of transmission, Iran’s government in March 2020 required all public members to stay at home, except for necessary purposes [4].

As a consequence of this pandemic, health workers are being faced with a heavy workload pressure, besides the increased total health expenditures. The immense burden of COVID-19 disease could cause caregiver burnout. Notably, the major sources of psychological distress among healthcare workers are as follows: increased work hours, lack of sleep quality, fatigue, and the risk of infecting with this virus and then putting their family members at the risk of a life-threatening condition [5]. Moreover, health care workers feel chronic fear of infection due to this virus’s contagious nature, unknown transmission modes, close contact with patients, and getting infection from their colleagues [6]. Recent research into the major sources of psychological distress among healthcare workers suggests that the well-being of the health care workforce is the basis of each well-functioning health system [7, 8]. Unfortunately, in Iran, at least 40 healthcare workers passed away due to COVID-19 infection and dozens have reportedly been under observation after presenting signs and symptoms of COVID-19 infection. Physicians’ burnout and lack of health care workforce have serious consequences for patients and could also lead the medical system to the verge of a devastating collapse [6].

In this study, we aimed to evaluate the impact of the COVID-19 epidemic on the workload and mental health of Iranian medical staff using the General Health Questionnaire (GHQ-12) and NASA-TLX Questionnaire between March and April 2020, respectively.

## Methods

### Participants and data collection

The present cross-sectional study was conducted from March 5th to April 5th, 2020. We targeted all of the health care workers such as nurses, doctors, emergency medical service staff, clinical, and public health technicians working in Iran ministry of health and medical education. We aimed for a convenience sample of participants. Informed written consent was obtained from all the participants included. Afterward, the anonymous online questionnaires were distributed among them. Accordingly, each health worker was allowed to fill the questionnaire for only one time.

### Questionnaires

#### Demographic questionnaire

This questionnaire included the subjects’ sociodemographic information such as age, marital status, sex, job title, shift working (fixed morning, fixed evening, fixed night or rotational), type of employment (contractual or permanent), over times per month (hrs.), duration of employment (in years), educational level (diploma, bachelor’s, master’s, doctoral, and higher), Governmental workplace (yes or no), having contact with COVID- 19 patients at workplace (yes or no), interest in job (yes or no), the increased working hours due to COVID-19 prevalence (yes or no), ward of work (ICU, operating room, laboratory, emergency, radiology, nursing station, COVID-19 service center, or others).

#### NASA-TLX questionnaire

To assess workload, we applied the NASA-TLX (NASA -Task Load Index) technique. Correspondingly, this technique was developed by the Human Performance Group at NASA Ames Research Center, which involved 6 subscales as follows: mental pressure, physical pressure, temporal pressure, performance, effort, and frustration. 20-step bipolar scales were then used to obtain ratings for these subscales. In this regard, the score of each scale was from 0 to 100. NASA-TLX score was also calculated by multiplying each subscale rate to its weight. Afterward, the overall workload was obtained by summing across scales and dividing by 15 [9, 10]. Mohammedi et al. in their study indicated the acceptable reliability of the NASA-TLX among health workers, with Cronbach’s alpha = 0.897 [11].

#### General Health Questionnaire (GHQ-12)

To evaluate the mental health (the psychosocial well-being), the General Health Questionnaire-12 (GHQ-12) was applied. Accordingly, GHQ was developed by Goldberg & Williams in 1972. Although this instrument initially had 60 items, currently there is a range of brief versions of the questionnaire including the GHQ-30, the GHQ-28, the GHQ-20, and the GHQ-12. Out of them, the GHQ-12 is short and easy to complete, and its application is appropriate in research settings. The GHQ-12 comprises of 12 items (six of which were positively phrased and six others were negatively phrased). Each item is rated on a 4-point scale (less than usual, no more than usual, rather more than usual, or much more than usual). Correspondingly, we used Goldberg’s original scoring method (0, 0, 1, and 1). This method supplies scores ranging from 0 to 12 [12]. Also, the appropriate reliability of Persian translation of the GHQ-12 was shown in a study by Montazeri et al. with Cronbach’s alpha = 0.87 [13].

### Statistical analysis

All statistical analyses were performed using IBM SPSS Statistics software. The normality of variables was confirmed using the Kolmogorov- Smirnov test. Moreover, chi-square test was used to compare the categorical data between the studied groups. The comparisons of the variables’ difference between the groups were performed using the independent Student’s t-test and ANOVA. Linear regression analysis in 3 models (Model 0: linear regression analysis without adjustment; Model I: linear regression analysis with adjustment for the encounter to coronavirus; and Model II: linear regression analysis with the correction of the encounter to the coronavirus, age, gender, marital status, job, experience, type of employment, shift, educational level, governmental, interested, and ward of work) was used for the determination of the association between overtimes of total Task Load score and GHQ score. Moreover, Spearman- test was used to indicate the correlation among overall Task Load score and NASA-TLX questionnaire components’ GHQ scores and age, educational level, and experience. A *p*-value of less than 0.05 was considered to be statistically significant.

## Results

In the present study, we analyzed 495 of the 1000 health workers who filled out the questionnaire, because 505 questionnaires were excluded from the study due to incomplete data. In terms of gender, 71.3% of the respondents were women. Also, the majority of respondents were nurses (65.9%). Regarding having contact with COVID- 19 patients at the workplace, 83.8% of respondents reported that they have contact with COVID- 19 patients. The participants’ characteristics in terms of the type of gender are shown in Table 1. In this regard, the differences in job, ward of work, and encountering COVID-19 patients were significant between women and men (*p* <  0.05). Moreover, men had significantly higher over time compared to women (76.57 ± 75. 87 vs. 58.49 ± 61.95, *p* = 0.01, respectively). (Table 1).

**Table 1:** The characteristics of the subjects in the male and female groups

Variable	Female (*n* = 353)	Male (*n* = 142)	*P-*value*
Education (N) (%)			
Doctoral degree and higher	9 (2.5)	9 (6.3)	0.058
Master	44 (12.5)	23 (16.2)	
Basic Sciences	259 (73.4)	89 (62.7)	
Diploma	41 (11.6)	21 (14.8)	
Age category (year), (N) (%)			
20–30	141 (39.9)	60 (42.3)	0.117
31–40	143 (40.5)	35 (36.6)	
41–50	64 (18.1)	23 (16.2)	
> 50	5 (1.4)	7 (4.9)	
Marital (N) (%)			
Single	133 (37.7)	43 (30.3)	0.073
Married	220 (62.9)	99 (69.7)	
Job (N) (%)			
Nurse	256 (72.5)	70 (49.3)	< 0.001
Doctor	32 (9.1)	11 (7.7)	
Health expert	6 (1.7)	9 (6.3)	
Health assistant	15 (4.2)	22 (15.5)	
Lab/radiology	6 (1.7)	8 (5.6)	
Other	38 (10.8)	22 (15.5)	
Experience (year), (N) (%)			
1–5	145 (41.1)	62 (43.7)	0.554
6–10	60 (17)	24 (16.9)	
11–15	77 (21.8)	22 (15.5)	
16–20	34 (9.6)	15 (10.6)	
> 20	37 (10.5)	19 (13.4)	
Type of employment (N) (%)			
Contractual	44 (12.5)	13 (9.2)	0.321
Permanent	137 (38.8)	47 (33.1)	
Employment contracts	86 (24.4)	40 (28.2)	
Temporary contracts	86 (24.4)	42 (29.6)	
Shift working (N) (%)			
Rotational	238 (67.4)	95 (66.9)	0.613
Night	10 (2.8)	2 (1.4)	
Morning	105 (29.7)	45 (31.7)	
Ward of work (N) (%)			
ICU	42 (11.9)	8 (5.6)	0.002
Operating room	18 (5.1)	8 (5.6)	
Laboratory	14 (4)	6 (4.2)	
Emergency	44 (12.5)	41 (28.9)	
Corona	45 (12.7)	13 (9.2)	
Radiology	13 (3.7)	5 (3.50	
Health center	140 (39.7)	47 (33.1)	
other	37 (10.5)	14 (9.9)	
Governmental workplace (N) (%)			
Yes	299 (84.7)	114 (80.3)	0.231
No	54 (15.3)	28 (19.7)	
Facing with COVID- 19 patients at workplace (N) (%)			
Yes	311 (88.1)	104 (73.2)	< 0.001
No	42 (11.9)	38 (26.8)	
Interest in job (N) (%)			
Yes	280 (79.3)	111 (78.2)	0.776
No	73 (20.7)	31 (21.8)	
Overtime (hour)	58.49 ± 61.95	76.57 ± 75. 87	0.01

The results are described as mean ± SD for quantitative data and number (%) for qualitative data

*P <  0.05 was considered as significant using Independent t-test for comparison between the two groups and Chi-square test for parametric and categorial data, respectively

As shown in Table 2, women had significantly higher GHQ scores compared to men (6.54 ± 1.84 vs. 5.90 ± 2.21, *p* = 0.003, respectively).

**Table 2:** Total Task Load score, NASA-TLX questionnaire components and GHQ score between the male and female groups

Variable	Female (*n* = 353)	Male (*n* = 142)	*P-*value*
Mental pressure	15.42 ± 4.25	14.7 ± 4.28	0.101
Physical pressure	13.79 ± 5.49	13.06 ± 5.55	0.218
Temporal	14.75 ± 4.48	13.69 ± 4.55	0.018
Performance	10.77 ± 7.01	12.66 ± 6.3	0.006
Effort	12.35 ± 6.17	13.8 ± 5	0.005
Frustration (failure)	14.2 ± 6.05	13.23 ± 6.01	0.110
NASA-TLX overall score	67.79 ± 17.85	68.95 ± 17.96	0.514
GHQ score	6.54 ± 1.84	5.90 ± 2.21	0.003

The results are described as mean ± SD. *P <  0.05 was considered as significant using Independent t-test for comparison between the two groups

*Abbreviation*: *NASA-TLX* NASA Task Load Index, *GHQ* General health Questionnaire

Total Task Load and GHQ scores according to different qualitative variables are presented in Table 3. Health workers who encountered COVID- 19 patients, were subjected to more task load and a lower GHQ score compared to those who had no contact with COVID- 19 patients at the workplace (*p* = 0.001). Notably, Total Task Load score was significantly higher in nurses compared to doctors and health assistants (71 ± 16.13 vs. 56.35 ± 20.45, *p* <  0.001; 71 ± 16.13 vs. 58.96 ± 15.28, *p* <  0.001). Furthermore, health experts had a higher task load compared to doctors (69.40 ± 8.85 vs. 56.35 ± 20.45, *p* = 0.012, respectively). The differences in Total Task Load scores were not significant among nurses and health experts (*p* = 0.999), radiology and laboratory experts (*p* = 0.868), and other jobs (*p* = 0.517). Regarding the ward of work, health workers of the Corona center had more total task load scores compared to the staff of health centers (71.56 ± 17.40 vs. 63.94 ± 17.36, *p* = 0.003). (Table 3).

**Table 3:** Total Task Load score and GHQ score according to different qualitative variables

Variables	Total Task Load score	GHQ score
Age category (year), (*n* = 495)		
20–30 (*n* = 201)	66.46 ± 18.16	6.10 ± 2.01
31–40 (*n* = 195)	68.96 ± 18.57	6.49 ± 2.11
41–50 (*n* = 87)	70.26 ± 15.38	6.49 ± 1.55
> 50 (*n* = 12)	66.75 ± 17.71	7.41 ± 0.51
*P- value*	0.32^*^	< 0.001^**a^
Marital (*n* = 495)		
Single (*n* = 176)	66.79 ± 17.52	5.98 ± 1.93
Married (*n* = 319)	68.31 ± 18.09	6.56 ± 1.96
*P- value*	0.744^*^	0.002^**^
Job (*n* = 495)		
Nurse (*n* = 326)	71 ± 16.13	6.43 ± 1.89
Doctor (*n* = 43)	56.35 ± 20.45	6.67 ± 1.98
Health expert (*n* = 15)	69.40 ± 8.85	6.73 ± 2.46
Health assistant (*n* = 37)	58.96 ± 15.28	5.62 ± 2.21
Lab/radiology (*n* = 14)	65.66 ± 20.41	6.78 ± 2.26
Other (*n* = 60)	66.82 ± 22.11	6 ± 1.93
*P- value*	< 0.001^**a^	0.076^*^
Marital Status		
Single(*n* = 176)	66.79 ± 17.52	5.98 ± 1.93
Married(*n* = 319)	68.31 ± 18.09	6.56 ± 1.96
*P- value*	0.744^***^	0.002^***^
Experience (year)		
1–5(*n* = 207)	66.97 ± 18.61	6.04 ± 2.04
6–10(*n* = 84)	68.97 ± 17.36	6.63 ± 1.96
11–15(*n* = 99)	69.54 ± 14.73	6.31 ± 2.14
16–20(*n* = 49)	72.18 ± 18.02	6.55 ± 1.55
> 20(*n* = 56)	65 ± 20.30	7.05 ± 1.48
*P- value*	0.240^*^	0.006^*a^
Type of employment		
Contractual(*n* = 57)	66.83 ± 18.25	6.91 ± 1.70
Permanent(*n* = 184)	69 ± 17.89	6.68 ± 1.84
Employment contracts(*n* = 126)	70.51 ± 18.40	6.15 ± 1.96
Temporary contracts(*n* = 128)	65.07 ± 16.87	5.86 ± 2.15
*P- value*	0.081^*^	< 0.001^**a^
Shift working		
Rotational(*n* = 333)	70.58 ± 17.13	6.39 ± 1.99
Night(*n* = 12)	75.22 ± 15.88	6.58 ± 1.44
Morning(*n* = 150)	61.50 ± 17.95	6.26 ± 1.96
*P- value*	< 0.001^*a^	0.741^*^
Education		
Doctoral degree and higher(*n* = 18)	68.15 ± 14.41	6.72 ± 2.27
Master (*n* = 67)	68.93 ± 16.8	5.67 ± 2.36
Basic Sciences (*n* = 348)	70.17 ± 16.23	6.40 ± 1.89
Diploma (*n* = 62)	55.76 ± 23.34	6.77 ± 1.74
*P- value*	< 0.001^**a^	0.008^*a^
Ward of work		
ICU(*n* = 50)	73.68 ± 16.22	6.50 ± 1.48
Operating room(*n* = 26)	82.32 ± 10.31	6.88 ± 1.17
Laboratory(*n* = 20)	69.11 ± 16.38	6.15 ± 1.72
Emergency(*n* = 85)	71.88 ± 16.38	6.83 ± 1.69
Corona center (*n* = 58)	71.56 ± 17.40	6.25 ± 1.91
Radiology(*n* = 18)	66.76 ± 16.98	7.33 ± 1.57
Health center(*n* = 187)	63.94 ± 17.36	5.90 ± 2.25
Other(*n* = 51)	60.70 ± 20.82	6.88 ± 2
*P- value*	< 0.001^*a^	< 0.001^**a^
Governmental workplace		
Yes(*n* = 413)	68.52 ± 17.89	6.47 ± 1.88
No(*n*^−42^)	66.11 ± 17.75	5.80 ± 2.30
*P- value*	0.265^***^	0.015^***^
Facing with COVID- 19 patients at workplace		
Yes(*n* = 415)	69.28 ± 17.50	6.52 ± 1.84
No(*n* = 80)	62.11 ± 18.68	5.53 ± 2.39
*P- value*	0.001^***^	0.001^***^
Interest in job		
Yes(*n* = 391)	67.11 ± 18.27	6.46 ± 1.99
No(*n* = 104)	61.93 ± 15.83	5.99 ± 1.85
*P- value*	0.015^***^	0.031^***^

Values are expressed as means ± SD

*P < 0.05 was considered as significant using One-way ANOVA test (F test). a. Post hoc with LSD test

**P < 0.05 was considered as significant using One-way ANOVA test (Welch test), a. Post hoc with LSD test

**P < 0.05 was considered as significant using Independent t-test for comparison between the two groups

*Abbreviation*: *NASA-TLX* NASA Task Load Index, *GHQ* General health Questionnaire

In terms of the subscale score of NASA-TLX, nurses had more scores in mental pressure, physical pressure, time pressure (temporal), and frustration compared to the other jobs (*p* <  0.05). Moreover, nurses had significantly more workload compared to the other jobs. (Table 4).

**Table 4:** Total Task Load score, NASA-TLX questionnaire components and GHQ score according to type of job

Variable	Nurse (*n* = 326)	Other (*n* = 169)	P-value*
Mental pressure	15.64 ± 3.94	14.40 ± 4.74	0.004
Physical pressure	14.85 ± 4.89	11.01 ± 5.78	< 0.001
Temporal	15.26 ± 4.04	12.89 ± 4.98	< 0.001
Performance	11.19 ± 6.76	11.54 ± 7.06	0.59
Effort	13.04 ± 5.73	12.30 ± 6.19	0.18
Frustration (failure)	15.15 ± 5.50	12.30 ± 6.19	< 0.001
NASA-TLX overall score	71.00 ± 16.13	62.57 ± 19.73	< 0.001
GHQ score	6.43 ± 1.89	6.21 ± 2.18	0.26

The results are described as mean ± SD. *P < 0.05 was considered as significant using Independent t-test for comparison between the two groups

*Abbreviation*: *NASA-TLX* NASA Task Load Index, *GHQ* General health Questionnaire

As shown in Table 5, total GHQ score had a significant positive correlation with age (*r* = 0.12, *p* = 0.008), educational level (*r* = 0.09, *p* = 0.03), and experience level (*r* = 0.15, *p* = 0.001). A positive significant correlation was also observed between mental pressure and age (*r* = 0.12, *p* = 0.007). In addition, a positive week significant correlation was observed between mental pressure and experience level (*r* = 0.10, *p* = 0.024). Notably, Task Load score, mental pressure, temporal, and performance had negative correlations with educational level (*p* <  0.05). (Table 5).

**Table 5:** The relationship between total Task Load score, NASA-TLX questionnaire components an GHQ score with age, Education, and Experience

Variables	Age category		Education level		Experience level	
	R	P- value*	R	P- value*	R	P- value*
GHQ score	0.12	0.008	0.09	0.03	0.15	0.001
Task Load score	0.07	0.098	− 0.12	0.005	0.04	0.387
Mental pressure	0.12	0.007	− 0.17	< 0.001	0.10	0.024
Physical pressure	− 0.06	0.179	0.01	0.777	−0.08	0.049
Temporal	−0.03	0.522	−0.10	0.023	−0.04	0.389
Performance	0.06	0.205	−0.13	0.004	0.05	0.236
Effort	0.05	0.224	−0.05	0.274	0.02	0.585
Frustration (failure)	−0.04	0.346	0.05	0.255	−0.03	0.536

*P < 0.05 was considered as significant using Spearman- test for correlation between variables. R was considered as correlation coefficient

*Abbreviation*: *NASA-TLX* NASA Task Load Index, *GHQ* General health Questionnaire

The relationship of overtime with total Task Load and GHQ scores is illustrated in Supplemental Table. In the unadjusted model, there was a significant association between Total Task load score and overtime (*B* = 0.025, *p* = 0.04), which did not remain significant after further adjustment for the encounter to COVID-19 patients (Model1), so it was adjusted for the encounter to the COVID-19 patients, age, gender, marital status, job, experience, employment status, shift, educational level, governmental workplace, interested in the job, and ward of work.(Supplemental Table).

## Discussion

In the present study, the workload and mental health levels affected by the COVID-19 outbreak were assessed among Iranian health care staff. More than 80% of the participants encountered COVID-19 patients in the workplace. Several variables such as age, marital status, experience, educational level, type of employment, ward of work interest in the job, and having contact with COVID-19 patients in the workplace had influences on the score of GHQ. Moreover, jobs, the shift of work, educational level, and facing COVID-19 affected the score of NASA-TLX. Generally, NASA-TLX scores were higher in nursing compared to other health staff groups. The results of this study indicated that the total workload and mental health levels of staff who treated COVID-19 patients were significantly worse than those who had no contact with COVID-19 patients. In a study by Lucchini et al., a 33% increase was indicated in the nursing workload among those who worked with COVID-19 patients in ICU. The authors suggested their colleagues worldwide to make an effort to increase the ICU nursing staff, to start training registered nurses from general wards to perform basic ICU procedures, and to dedicate intensive care nurses to manage more complex procedures, in order to be prepared to face the epidemic [14]. During the COVID-19 pandemic, it was shown that healthcare workers are at a higher risk of exposure, so the application of personal protective equipment (PPE) is necessary. Accordingly, the mandatory use of PPE dramatically elevates both nursing workload and fatigue [15]. Achieving a sufficient health care workforce during this infection epidemic not only needs a sufficient number of health care providers, but also maximizes the ability of each clinician in caring for a high volume of patients [16]. Cao et al. in their study concluded that the hospital emergency management plan of West China Hospital could reduce the emergency department (ED) workload, protect healthcare staff, and control the cross-infection during the COVID-19 epidemic. Additionally, they approved that each hospital should establish a specific contingency plan according to its condition [17].

Few studies have been conducted on the physical and psychological effects of outbreaks of serious infectious diseases among the medical staff, particularly when they have increased workload and the stress associated with the risk of infection [18]. Liu et al. conducted a qualitative study on nurses and physicians who were selected from five COVID-19-designated hospitals in Hubei province. In line with our findings the authors indicated that intensive work drains healthcare providers both physically and emotionally. Healthcare providers showed their resilience as well as a great strength of professional dedication to overcome problems. The authors suggested that a comprehensive support should be supplied to protect the well-being of healthcare providers. Also, a regular and intensive training plan for all healthcare providers is necessary to promote their preparedness and efficacy to deal with crises [19].

The current study showed that workload and shift working had a significant association with each other, and night shift had higher workload scores compared to rotational and morning shifts. Accordingly, these findings are consistent with the findings of the Hoonakker et al.’s study. They showed that night shifts had a higher workload compared to the morning shift. Also, their study showed that shifts with an 8 h cycle time had a lower mental workload in comparison with a 12-h shift time [20]. So, shortening work shifts and adjusting shifts to psychophysiological characteristics workers can improve worker performance to manage crisis [21, 22].

The limitations of this study were as follows: firstly, the sample composition was uneven. Moreover, a lack of response to the questionnaire due to potential bias like the COVID-19 crisis in responding to questionnaires, not assessing the income of healthcare workers, and having any other disease were the other limitations of the present study.

## Conclusions

Type of job, the shift of work, educational level, and facing COVID-19 affected the score of NASA-TLX. Generally, NASA-TLX scores were higher in nursing compared to the scores of other health staff groups. The results of this study indicate that the scores of NASA-TLX and GHQ-12 among staff who had contact with COVID-19 patients were significantly higher than those who did not face COVID-19 patients. We suggested that a comprehensive assistance should be provided to support the well-being of healthcare workers especially nurses and healthcare workers who treated COVID-19 patients.

## Supplementary Information

**Additional file 1: Supplemental Table.** The relationship between overtime with total Task Load score and GHQ score (dependent variables).

## Data Availability

The datasets used and/or analyzed during the current study are available from the corresponding author on reasonable request.

## Abbreviations

HCW_s_: Health care workers
PHEIC: Public Health Emergency of International Concern
IOM: Institute of Medicine
WHO: World health organization
AIDS: Acquired immunodeficiency syndrome
NASA-TLX: NASA -Task Load Index
GHQ-12: General health Questionnaire
ANOVA: Analysis of variance
ICU: Intensive care unit
COVID-19: Coronavirus disease 2019
