# Supplementary material for: Covid-19 effects on the workload of Iranian healthcare workers
Source: BMC Public Health. 2020 Nov 2;20:1636. doi: 10.1186/s12889-020-09743-w (PMC7605333; doi:10.1186/s12889-020-09743-w)
Supplement: Supplementary file 1 — Additional file 1: Supplemental Table. The relationship between overtime with total Task Load score and GHQ score (dependent variables). [file 12889_2020_9743_MOESM1_ESM.docx]

**Supplemental Table.** The relationship between overtime with total Task Load score and GHQ score (dependent variables).

| Crude model | B | R Square | (95% CI) | P-value* |
| --- | --- | --- | --- | --- |
| Total Task Load score | 0.025 | 0.008 | (0.001, 0.04) | 0.04 |
| TGHQ score | 0.002 | 0.003 | (-0.001, 0.004) | 0.19 |
| Model 1 |  |  |  |  |
| Total Task Load score | 0.023 | 0.02 | (<0.001, 0.04) | 0.054 |
| TGHQ score | 0.25 | 0.03 | (-0.001,.004) | 0.25 |
| Model 2 |  |  |  |  |
| Total Task Load score | 0.01 | 0.13 | (-0.01, 0.03) | 0.38 |
| TGHQ score | 0.001 | 0.12 | (-0.001,0.004) | 0.29 |

* *P<0.05* statistically significant by linear regression. Model 0(Crude model): unadjusted, Model 1: adjusted for encounter to the COVID-19 patients, Model 2: adjusted for encounter to the COVID-19 patients, age, sex, marital, job, experience, employment status, shift, education, governmental workplace, interested in job, and ward of work.

**Abbreviation**: NASA-TLX: NASA Task Load Index, GHQ: General health Questionnaire
